# The Trypomastigote Small Surface Antigen (TSSA) regulates *Trypanosoma cruzi* infectivity and differentiation

**DOI:** 10.1371/journal.pntd.0005856

**Published:** 2017-08-11

**Authors:** María de los Milagros Cámara, Gaspar E. Cánepa, Andrés B. Lantos, Virginia Balouz, Hai Yu, Xi Chen, Oscar Campetella, Juan Mucci, Carlos A. Buscaglia

**Affiliations:** 1 Instituto de Investigaciones Biotecnológicas-Instituto Tecnológico de Chascomús (IIB-INTECh), Universidad Nacional de San Martín (UNSAM) and Consejo Nacional de Investigaciones Científicas y Técnicas (CONICET), Buenos Aires, Argentina; 2 Instituto de Tecnología, Universidad Argentina de la Empresa (UADE), Buenos Aires, Argentina; 3 Department of Chemistry, University of California, Davis, Davis, California, United States of America; Harvard School of Public Health, UNITED STATES

## Abstract

**Background:**

TSSA (Trypomastigote Small Surface Antigen) is an antigenic, adhesion molecule displayed on the surface of *Trypanosoma cruzi* trypomastigotes. *TSSA* displays substantial sequence identity to members of the *TcMUC* gene family, which code for the trypomastigote mucins (tGPI-mucins). In addition, TSSA bears sequence polymorphisms among parasite strains; and two TSSA variants expressed as recombinant molecules (termed TSSA-CL and TSSA-Sy) were shown to exhibit contrasting features in their host cell binding and signaling properties.

**Methods/Principle findings:**

Here we used a variety of approaches to get insights into TSSA structure/function. We show that at variance with tGPI-mucins, which rely on their extensive *O*-glycoslylation to achieve their protective function, TSSA seems to be displayed on the trypomastigote coat as a hypo-glycosylated molecule. This has a functional correlate, as further deletion mapping experiments and cell binding assays indicated that exposition of at least two peptidic motifs is critical for the engagement of the ‘adhesive’ TSSA variant (TSSA-CL) with host cell surface receptor(s) prior to trypomastigote internalization. These motifs are not conserved in the ‘non-adhesive’ TSSA-Sy variant. We next developed transgenic lines over-expressing either TSSA variant in different parasite backgrounds. In strict accordance to recombinant protein binding data, trypomastigotes over-expressing TSSA-CL displayed improved adhesion and infectivity towards non-macrophagic cell lines as compared to those over-expressing TSSA-Sy or parental lines. These phenotypes could be specifically counteracted by exogenous addition of peptides spanning the TSSA-CL adhesion motifs. In addition, and irrespective of the TSSA variant, over-expression of this molecule leads to an enhanced trypomastigote-to-amastigote conversion, indicating a possible role of TSSA also in parasite differentiation.

**Conclusion/Significance:**

In this study we provided novel evidence indicating that TSSA plays an important role not only on the infectivity and differentiation of *T*. *cruzi* trypomastigotes but also on the phenotypic variability displayed by parasite strains.

## Author summary

Infection with *Trypanosoma cruzi* produces a chronic and debilitating infectious disease known as Chagas disease, of major significance in Latin America and an emergent threat to global public health. In the absence of vaccines and/or appropriate chemotherapies, the search for parasite effectors that support infection of mammalian cells is a focus of significant interest. One such candidate is the Trypomastigote Small Surface Antigen (TSSA), a polymorphic molecule expressed on the surface coat of infective trypomastigote forms. Previous data indicated that recombinant versions of two different TSSA variants (termed TSSA-CL and TSSA-Sy) encoded by parasite strains belonging to extant phylogenetic groups exhibited contrasting host cell binding and signaling abilities. Here, we generated genetically modified strains of *T*. *cruzi* over-expressing different TSSAs to address this issue. Trypomastigotes over-expressing TSSA-CL, the ‘adhesive variant’, displayed improved adhesion and infectivity towards non-macrophagic cell lines as compared to those over-expressing TSSA-Sy or parental lines. In addition, and irrespective of the protein variant, TSSA over-expression enhanced trypomastigote-to-amastigote conversion. Overall, our data strongly suggest that TSSA plays an important role not only on the infectivity and differentiation of *T*. *cruzi* trypomastigotes but also on the phenotypic variability displayed by different strains of this parasite. These data, together with the fact that TSSA recalls a strong and likely protective humoral response during human infections, support this molecule as an excellent candidate for molecular intervention and/or vaccine development in Chagas disease.

## Introduction

*Trypanosoma cruzi* is the protozoan agent of Chagas disease, of major medical and economic significance throughout Latin America, and an emergent threat to global public health [1]. This parasite alternates between blood-sucking triatomine vectors and a wide spectrum of susceptible, mammalian hosts, including humans. Transitions between hosts or from different niches within hosts pose major adaptation challenges and are accordingly accompanied by a complex series of developmental changes [2]. Briefly, epimastigote forms replicate along the digestive tract of insect vectors, and eventually attach to the cuticle of the rectal epithelium where they differentiate into non-dividing and infective metacyclic trypomastigotes. These are in turn deposited on the mammal along with the insect feces during a blood meal and gain access to internal body fluids via a skin lesion or a mucosal surface. In the mammalian host, *T*. *cruzi* presents two major morphological stages: trypomastigotes, which are non-dividing and highly motile forms found in the bloodstream and in the extracellular spaces of tissues, and amastigotes, which are intracellular replicative forms.

Host cell invasion by *T*. *cruzi* trypomastigotes follows a rather complex, active and multi-step process [3,4]. The initial recognition and sensitization of the target cell involves various apparently redundant parasite surface receptors such as MASPs (mucin-associated surface proteins) and Gp85 molecules [5–7]. Most of these are glycosyl phosphatidylinositol (GPI)-anchored glycoproteins encoded by large, polymorphic and developmentally-regulated gene families [6,8]. They all bear a similar predicted architecture, in which the outermost and variable *N*-terminal domain protrudes from the parasite glycocalix, thus ideally suited for engaging with different constituents of the host cell membrane and/or extracellular matrix. Early after invasion, trypomastigotes escape from the parasitophorous vacuole into the host cell cytoplasm where they undergo transformation into amastigote forms. The molecular and cellular mechanisms that regulate amastigogenesis remain poorly understood, although this process can be triggered and recapitulated, at least *in vitro*, by various factors including temperature or pH shift and starvation [9,10]. After several rounds of replication and just before disruption of the parasite-laden cell, amastigotes differentiate back into trypomastigotes, which may infect other cells within the host or may be taken up by the insect vector during a bloodmeal, thus closing the cycle.

*T*. *cruzi* trypomastigotes are wrapped by a protective coat made up essentially of heavily *O*-glycosylated mucins and, underneath, glycoinositol phospholipids (GIPLs) [11,12]. Trypomastigote mucins, also known as tGPI-mucins, are encoded by the *TcMUC* family of genes, comprising ~800 members [13–15]. *TcMUC* deduced products share a common structure made up of highly conserved *N*-terminal signal peptide (SP) and cleavable *C*-terminal GPI attachment signal; and a variable, Thr-rich region that constitutes the ‘mature’ apo-mucin displayed on the parasite surface [13]. The latter region undergoes extensive *O*-glycosylation *in vivo*, which confers a strong hydrophilic character to the overall molecule [11,16]. Large variability observed for biochemically-purified tGPI-mucins may be attributed to the simultaneous expression of multiple *TcMUC* genes showing differences in the length, sequence and number of putative glycosylation-acceptor sites as well as in the extent and/or structure of attached oligosaccharides [13–17]. *O*-glycans in tGPI-mucins start with the addition of a single αN-acetylglucosamine (αGlcNAc) unit to the hydroxyl group of Thr residues, which may remain unsubstituted or become elongated with different carbohydrates (mainly Galactopyranoses, Gal*p*) in multiple linkages and configurations [16,18]. In the presence of suitable donors, tGPI-mucins become rapidly sialylated by means of a parasite-encoded *trans*-sialidase (TS) [11,18–20]. This reaction involves the cleavage of a sialic acid (SA) residue linked in α(2–3) configuration to a terminal βGal*p* (SAα2–3βGal*p*) in the donor macromolecule and the subsequent formation of the same linkage on tGPI-mucins [18,21]. Alternatively, certain βGal*p* terminal units of tGPI-mucins may become modified with αGal*p* residues within the parasite secretory pathway, which generates highly antigenic structures and precludes their further elaboration with SA on the trypomastigote membrane [22]. Importantly, SA-containing neo-glycotopes on tGPI-mucins are involved in trypomastigote recognition and invasion of mammalian cells [23,24], as well as in providing protection against lytic antibodies [11] and/or complement opsonization [25]. In addition to their surface-associated roles, tGPI-mucins are also shed to the milieu as part of multi-cargo and multi-tasking micro-vesicles (MVs) that bud from the trypomastigote plasma membrane [26,27]. The lipid anchor of tGPI-mucins triggers the synthesis of proinflammatory cytokines in macrophages and other immunomodulatory phenomena in the infected host [28–30].

TSSA (Trypomastigote Small Surface Antigen) is a highly antigenic GPI-anchored molecule expressed by *T*. *cruzi* trypomastigotes, likely involved in its interaction with host cells [31–35]. *TSSA* displays substantial sequence conservation to members of the *TcMUC* family, particularly along its deduced *N*- and *C*-terminal regions, and was thus included within this family [15,32]. However, *TSSA* codes for a short polypeptide with no Thr-rich region that migrates in non-reducing SDS-PAGE as a single ~15 kDa species [32], thus well below the molecular mass determined for tGPI-mucins (~45–220 kDa [16]). Also at variance with tGPI-mucins, TSSA adhesion properties seem to rely on the exposition of peptidic rather than glycan moieties [23,31]. This point was further assessed by direct binding assays of recombinant TSSA molecules to cultured mammalian cells [31]. Detailed genetic characterization of the *TSSA* locus disclosed sequence variations among parasite strains that, when considered as a whole, turned out to be diagnostic for each of the 6 phylogenetic groups (named as TcI to TcVI) delineated within the *T*. *cruzi* taxon [36,37]. Interestingly, two TSSA variants expressed by TcI and TcVI extant *T*. *cruzi* clades were shown to exhibit significant differences in their antigenicity as well as in their host cell binding and signaling properties [31,32,38].

Here we show that although encoded by *TcMUC* genes and being co-expressed on the trypomastigote coat, TSSA and tGPI-mucins are completely different species. While tGPI-mucins undergo extensive *O*-glycosylation and terminal sialylation to fulfill their main protective roles, TSSA seems to be displayed as a hypo-glycosylated molecule. Both kind of molecules segregate to mutually exclusive membrane domains, further supporting their functional diversification. We next used a variety of biochemical and genetic approaches to demonstrate that exposition of ‘naked’ peptidic motifs is critical for TSSA engagement to host cell surface receptor(s) prior to trypomastigote internalization.

## Materials and methods

### Parasite stocks and cell lines

CL Brener and Sylvio X-10 clones, belonging to TcVI and TcI *T*. *cruzi* phylogenetic groups, respectively, were used in this study. Parasite developmental forms were obtained and purified as described [39,40]. Briefly, epimastigote forms were grown at 28°C in brain-heart tryptose (BHT) medium (BD) supplemented with 10% fetal calf serum (FCS, GIBCO Laboratories) whereas cell-derived trypomastigotes (henceforth trypomastigotes) and amastigotes were harvested from the supernatant of infected, *Mycoplasma*-free monkey fibroblast Vero cells (ATCC). Vero and human HeLa cells (ATCC) were grown at 37°C and 5% CO_2_ in DMEM supplemented with 10% FCS, 0.292 g/L L-glutamine, 100 IU/mL Penicillin and 100 μg/mL Streptomycin (all from GIBCO Laboratories).

### Parasite transfection

Tagged versions of the TSSA variant encoded in the Esmeraldo-like chromosome of the CL Brener clone (TSSA-CL; GenBank Accession Number ACY54510), and the TSSA variant encoded by the Sylvio X-10 clone (TSSA-Sy; GenBank Accession Number ACY02865.1) have been described [31]. The tagged version of a canonical *TcMUC* gene (GenBank Accession Number U32448) has also been described [41]. All of these constructs bear a single, in-frame FLAG epitope placed immediately upstream of the GPI-anchoring signal, which does not interfere with protein trafficking, processing and/or surface display [31,41]. These constructs were subcloned into the episomal *pTEX-omni* plasmid [42] by using the XbaI and XhoI restriction sites. Cloning was checked by restriction mapping analysis and DNA sequencing. For parasite transfection, exponentially growing epimastigotes (3 x 10^8^) were harvested, washed with phosphate-buffer saline (PBS), transferred to a 0.2 cm gap cuvette (Bio-Rad) with 10 μg of purified DNA and electroporated as described [42]. Parasites were not cloned by limited dilution or enriched by any means, and antibiotic selection (500 μg/mL G418; GIBCO Laboratories) was sustained over time once stable transfected populations were obtained. For growth curves, epimastigotes were seeded at a density of 1 x 10^6^ parasites per mL in BHT 10% FCS without G418 and the cell number was quantified at the indicated time-points using a Neubauer chamber. For metacyclogenesis assays exponentially growing epimastigote forms (2 x 10^6^) were harvested, diluted in BHT without G418 and maintained at 28°C without agitation. Metacyclogenesis was evaluated at different time-points by direct counting on Neubauer chambers. For each sample, at least 150 fixed parasites were counted and epimastigote vs metacyclic forms were discriminated morphologically [2].

### Parasite fractionation, Western blot, and flow cytometry analyses

Total parasite lysates were run in SDS-PAGE, transferred to PVDF membranes (GE Healthcare), and probed with mouse monoclonal antibody (mAb) anti-FLAG (clone M2, Sigma) followed by HRP-conjugated anti-mouse IgG (Sigma) (both at 1:5,000 dilution) and the SuperSignal West Femto Chemiluminescent Substrate (Pierce). Antiserum to TSSA-CL [31,32], to TcMUC [19], to *T*. *cruzi* TcSMUG L [43] and to *T*. *cruzi* Glutamate Dehydrogenase (TcGDh, [44]) were used at 1:5,000, 1:2,000, 1:5,000 and 1:5,000 dilution, respectively. Western blots on total parasite extracts were performed to assess the recognition protein profiles for antisera to TcMUC and TSSA-CL (S1 Fig). For flow cytometry analyses, parasites (1.5 x 10^6^) were washed, blocked in PBS 10% FCS, and incubated with rabbit polyclonal antibodies to FLAG (Sigma) or antiserum to TSSA-CL (both at 1:200 dilution), in an ice-water bath followed by Alexa Fluor-conjugated secondary antibodies (1:500 dilution) (Molecular Probes). After several washes, parasites were resuspended in 300 μL of PBS containing 4% (w/v) paraformaldehyde (PFA), extensively washed with PBS and analyzed using FACS CyFLOW Partec and FloMax software.

### Indirect immunofluorescence (IIF) assays

Parasites were harvested, washed in PBS, adhered to poly-*L*-lysine (Sigma) coated cover-slips, fixed for 30 min in PBS 4% PFA, blocked for 30 min in 4% Bovine Serum Albumin (BSA, Sigma) in PBS (PBS-BSA) supplemented with 0.5% saponin (Sigma) for permeabilization, and probed with mAb anti-FLAG diluted 1:500 in PBS-BSA or rabbit antiserum to TSSA-CL (1:4,000 dilution). After extensive washings with PBS, secondary Alexa Fluor-conjugated antibodies were added at 1:500 dilution in PBS-BSA. Samples were extensively washed with PBS and mounted. Images were obtained with a Nikon Eclipse 80i epi-fluorescence microscope coupled to a DS-Qi1 CCD camera or with an IX-81 microscope attached with a FV-1000 confocal module. In the latter case, the objective was a PLAN APO 60X NA 1.42 oil immersion (Olympus, Japan) and the acquisition software used was FV 10-ASW 3.1. Images were treated using ImageJ 1.45s Software (NIH, USA).

### *In vitro* adhesion and infection assays

Vero cells (5 x 10^4^) grown on 24-well culture plates were added with 1 or 2 x 10^5^ Sylvio X-10 or CL Brener trypomastigote forms (with up to 5% of contaminant amastigote forms), respectively. When indicated, parasites were mixed with an equal volume of a specific peptide at 100 μg/mL or PBS before being added to the cell monolayer. After 3 h of incubation at 4°C (for adhesion assays) or at 37°C (for infection assays), cells were washed with PBS to remove non-attached parasites and fixed with PBS-PFA immediately (for adhesion assays) or after additional 36 h incubation in DMEM 4% FCS at 37°C (for infection assays). Following extensive washings in PBS, cells were processed for IIF assays as described [31]. Infection rate was determined by manual counting of infected and total cells whereas adhesion rate was determined by counting cells with adhered/recently internalized parasites and total cells using the Image J plug-ins Cell Counter and Nucleus Counter in at least 1,000 DAPI-stained cells. Three independent experiments were carried out, each one in duplicate.

### Trans-well infection assays and preparation of parasite conditioned medium

Vero cells (1 x 10^4^) were seeded on the bottom of trans-well microplates (0.4 mm pore size, Corning Fisher, NY). After 24 h cells were added with 1 x 10^5^ wild type trypomastigotes whereas 2 x 10^5^ trypomastigotes of the indicated transgenic or parental line were seeded in the upper chamber of the trans-well. Following a 3 h-incubation period at 37°C, the upper trans-well chambers were removed, cells were washed with PBS to remove non-attached parasites and processed for IIF assays after additional 36 h incubation in DMEM 4% FCS. Parasite conditioned medium (CM) was prepared as described [19] and diluted 1:2 in fresh MEM 4% FCS for infection assays. When indicated, CM was fractionated onto 25 μL of mAb anti-FLAG-Sepharose (Sigma) or control anti-hemmaglutinin-Agarose (Roche) as described [43]. Flow-through (unbound) fractions were then used for infection assays as above.

### Protein binding assays

Glutathione *S*-transferase (GST) fusion proteins bearing the central and mature region of TSSA-Sy (GST-TSSA-Sy^24-61^) and TSSA-CL (GST-TSSA-CL^24-62^), as well as variants spanning partially overlapped sequences from TSSA-CL^24-62^ have been described [33]. HeLa cells (5 x 10^4^) placed in 96-well culture plates were grown overnight, fixed with PBS 4% PFA, blocked with PBS 10% FCS for 1 h, and incubated with 200 μg/mL of the indicated GST-fusion protein for 1 h followed by mAb anti-GST (clone GST-2, Sigma) diluted 1:1,000 in PBS 2% FCS. Plates were washed with PBS, added with HRP-conjugated secondary antibody diluted 1:5,000 in PBS 2% FCS followed by 100 μL of 3,3’,5,5’-Tetramethylbenzidine and 50 μL 2 M sulfuric acid, and the absorbance read at 450 nm.

### Synthetic peptides

Custom peptides were synthesized by GenScript. Sequences were as follows: pCL22-38, ^22^CTTANGGSTSSTPPSGT^38^; pCL30-44, ^30^TSSTPPSGTENKPAT^44^; pCL42-56, ^42^PATGEAPSQPGASSG^56^; and pSy41-55, ^41^TAAGGTPSPSGASSG^55^. These were derived from TSSA-CL or TSSA-Sy deduced protein sequences; and residues are numbered according to their position in the corresponding protein. Neither peptide affected parasite and/or cell viability under the assayed conditions, as revealed by propidium iodide uptake and trypan blue exclusion, respectively [45].

### *In vitro* amastigogenesis assays

Recently harvested trypomastigotes (5 x 10^6^) were washed twice in PBS supplemented with 2% glucose and incubated for 48 h in MEM without FCS at either pH7 or pH5, at 37°C. Samples were taken at the indicated time-points, washed twice with PBS and fixed with PBS-PFA 4%. Direct counting of cell-derived trypomastigote and amastigote forms were determined on Neubauer chambers. Alternatively, amastigogenesis was assessed by IIF assays, labeling parasite samples taken at the indicated time-points with an antiserum raised against a fragment (residues 20 to 67) of an amastin protein (TcCLB.506437). The use of amastin as a *T*. *cruzi* amastigote marker has been validated [46].

### Sialic acid labeling of *T*. *cruzi* trypomastigotes

Live trypomastigote forms from the CL Brener strain were extensively washed in cold PBS and labeled for 30 min in the presence of 10 mM 2-deoxyglucose (Sigma) and 1 mM of the azido-sialyllactose analog *N*-azidoacetyl neuraminyl α2–3lactose (Neu5Azα2–3LacβOMe) [19]. When indicated, recombinant TS was added to the reaction mixture as described [43]. Reaction was heated at 65°C to inactivate TS and non-permeabilized parasites labeled by the Staudinger method with 250 μM Phosphino-FLAG (Sigma) for 20 min at room temperature [19]. Following extensive washings, parasites were processed for IIF assay as above or resuspended (at 500 x 10^6^ per mL) in ice-cold immunoprecipitation buffer (150 mM NaCl, 50 mM Tris/HCl, pH 7.6, 1 mM EDTA, 0.1% Nonidet P40, 1% Triton X-100, 100 μM Tos-Lys-CH_2_Cl and 1 mM PMSF) and incubated on ice for 1 h. After preclearing, samples were fractionated onto 25 μL of mAb anti-FLAG-Sepharose overnight at 4°C. Following several washings, retained molecules were directly cracked in 100 μL for Western Blot. Unbound fractions were precipitated with acetone and processed for Western blot.

### Mucin purification

Trypomastigote forms (1–3×10^9^) were delipidated by chloroform/methanol/water (10:20:8 v/v/v) treatment as described [16]. Briefly, the soluble fraction was evaporated under N_2_ stream and then partitioned with butan-1-ol/water (2:1, v/v). The butan-1-ol phase (F1) contains mainly lipids, phospholipids and GIPLs, whereas the aqueous phase (F2) is enriched in mucins. Both phases were further extracted as before. Delipidated parasite pellets were also extracted with butan-1-ol/water (2:1, v/v) at 4°C, and the mucin-rich aqueous phase (F3) was stored. Final parasite pellets (P) were resuspended in denaturing loading buffer containing 6 M urea and 100 μg/mL DNAse I (Sigma).

### ß-elimination

Epimastigotes (2×10^8^) were harvested, washed with PBS, resuspended in 200 μL of lysis buffer (Tris-HCL 20 mM pH 7.6, EDTA 1mM, Sacarose 0,25M, Nonidet-P40 0,5% (v/v)) and incubated on ice for 1 h. Upon centrifugation to remove cellular debris supernatants were added with NaOH (0.1 N final) and incubated for 4 h at 40°C. Treated and untreated samples were analyzed by Western Blot.

### Micro-vesicle harvest of trypomastigote conditioned medium

CL Brener trypomastigotes (2 × 10^8^) were subjected to four consecutive centrifugation rounds, two at 2,700 × *g* for 10 min followed by two at 10,600 × *g* for 10 min. The cell-free CM was supplemented with 10% FCS to a final volume of 100 μL. Exosome purification kit (System Biosciences, CA) was added to the CM and MVs were purified according to the manufacturer’s protocol. The pellet containing MVs was cracked in a final volume of 100 μL while proteins in the supernatant were precipitated with cold acetone, cracked in a final volume of 100 μL and analysed by Western blot [19].

## Results

### TSSA and tGPI-mucins constitute different species on the trypomastigote coat

**Fig 1:**
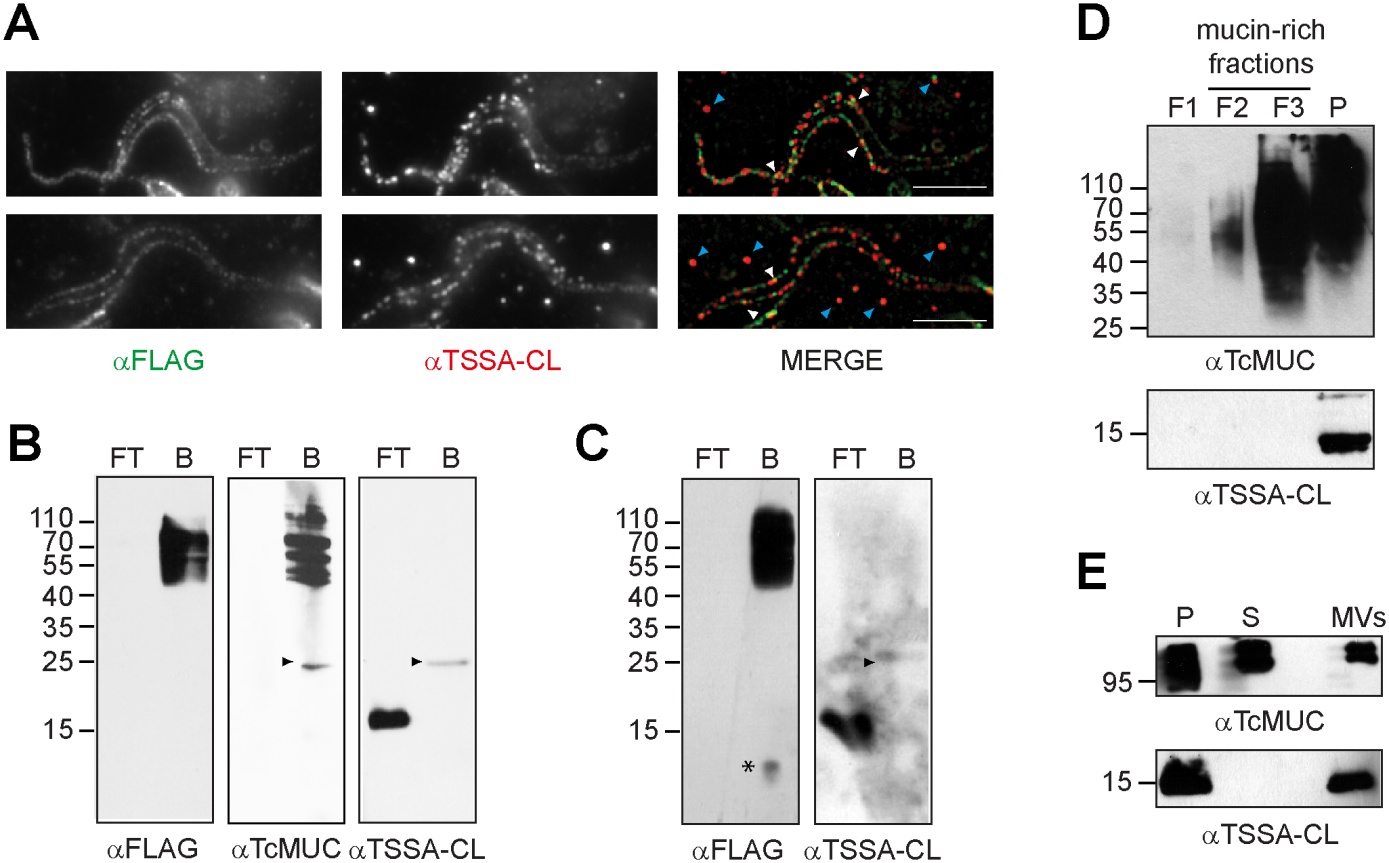
TSSA is not a sialic acid acceptor and does not behave as a typical *T*. *cruzi* mucin. **A)** Representative images of CL Brener trypomastigotes incubated with Neu5Az*α*2-3Lac*β*OMe and processed for immunofluorescence using both mouse mAb anti-FLAG (green) and rabbit TSSA-CL antiserum (red). White arrowheads point to few co-localization spots. Cyan arrowheads indicate TSSA-CL-reactive particles probably secreted to the medium. Bars = 10 μm. **B** and **C)** Intact CL Brener trypomastigotes were labeled with Neu5Az*α*2-3Lac*β*OMe in the absence (panel B) or presence (panel C) of recombinant *trans*-Sialidase. The labeled material was fractionated on to anti-FLAG–Sepharose and flow-through (FT) and bound (B) fractions were probed by Western blot. Arrowheads point to a ~25 kDa band, which likely represents the light chain of the anti-FLAG mAb that leaked out of the anti-FLAG-Sepharose. A faint ~10 kDa band of unknown identity is denoted with an asterisk in panel C. **D)** Butan-1-ol extraction analysis of CL Brener trypomastigotes. Fractions were obtained according to Materials and Methods, and processed for Western blot. **E)** Conditioned medium from CL Brener trypomastigotes was fractionated using Exoquick kit. Fractions corresponding to parasite pellet (P), soluble molecules (S) and micro-vesicles (MVs) were analyzed by Western blot. Relative molecular mass markers (in kDa) are indicated.

One key and defining functional aspect of tGPI-mucins is their role as major SA acceptors on the trypomastigote coat [11,19,23]. Hence, to evaluate if TSSA may be functionally considered part of tGPI-mucins, we firstly analyzed the distribution of SA-containing glycoconjugates and TSSA on the parasite surface by fluorescence microscopy. To that end, CL Brener trypomastigotes were sialylated in the presence of exogenously added Neu5Azα2-3LacβOMe. This sialyllactose analog is recognized as an appropriate SA residue donor by *T*. *cruzi* TS and readily incorporated into the trypomastigote coat [19]. Once incorporated, the azido group of Neu5Az was covalently coupled to a FLAG epitope through a Cu^2+^-free click chemistry, thus allowing us to assess the distribution of SA-acceptors by anti-FLAG IIF assays. As shown in Fig 1A, the surface of trypomastigotes became strongly labeled upon addition of Neu5Azα2-3LacβOMe. Control IIF assays carried out in the absence of Neu5Az*α*-3Lac*β*OMe or over heat-killed parasites rendered negative results [19], indicating the requirement of active TS for effective incorporation of the derivative sialyl residue.

As described [19], SA-acceptors were displayed in discrete domains following a dotted pattern along the entire surface of the parasite body and the flagellum (Fig 1A). TSSA also presented a discontinuous distribution, with multiple anti-TSSA-reactive spots of apparent larger size than those of SA-acceptors scattered along the trypomastigote surface (Fig 1A). Additional TSSA-reactive spots were observed in the vicinity of trypomastigotes, which may correspond to secreted TSSA molecules (Fig 1A). Importantly, minimal co-localization was observed between TSSA and SA-acceptors signals (Fig 1A). We next fractionated Neu5Az-labeled glycoconjugates on to mAb anti-FLAG-Sepharose and evaluated different fractions by Western blot. As shown, SA-acceptors migrated in reducing SDS-PAGE as a broad smear ranging from 115 to 45 kDa (Fig 1B, left panel), a pattern compatible with tGPI-mucins [16,19]. Indeed, part of the Neu5Az-labeled material also reacted with an antiserum raised against a canonical TcMUC product (Fig 1B, middle panel). In contrast, the ~15 kDa TSSA-reactive band was observed exclusively in the flow-through (i.e. non-sialylated) fraction (Fig 1B, right panel). Further labeling experiments carried out in the presence of recombinant TS (to disregard possible steric hindrance and/or enzyme shortage that would interfere with TS-TSSA interaction in our previous experimental setup) yielded similar results (Fig 1C). In such conditions, and in addition to the 45–115 kDa smear of tGPI-mucins we were also able to detect a faint ~10 kDa FLAG-reactive band in the bound fraction (Fig 1C, left panel), which was not further characterized. Again, TSSA partitioned completely to the unbound fraction (Fig 1C, right panel). Together, these results strongly suggest that TSSA molecules displayed on the trypomastigote surface do not constitute significant SA-acceptors for parasite TS *in vivo*. This may in turn reflect the absence of terminal βGal*p* residues in appropriate configuration on TSSA or, alternatively the presence of specific modifications (i.e. α-galactosylation) on such terminal residues that preclude their further conjugation to SA by means of TS [16]. To distinguish between these possibilities, we next followed a standard butan-1-ol extraction protocol in unlabeled CL Brener trypomastigotes and analyzed different fractions by Western blot. As shown in Fig 1D, TSSA is detected exclusively in the parasite-associated fraction, strongly suggesting that it does not undergo extensive glycosylation *in vivo*. In contrast, The TcMUC antiserum revealed a broad smear in the aqueous soluble fractions F3 and, to a lesser extent, F2, which coincides with the extraction pattern of sialylated or α-galactosylated tGPI-mucins (Fig 1D) [16,19]. We finally evaluated the pattern of spontaneous secretion of TSSA and tGPI-mucins by CL Brener trypomastigotes. As shown in Fig 1E, secreted TSSA is entirely associated to plasma membrane-derived MVs whereas at least part of secreted tGPI-mucins partitioned to the ‘soluble’ fraction of the conditioned medium (CM) of trypomastigotes (Fig 1E). Overall, these findings indicate that TSSA and tGPI-mucins display substantial biochemical divergences and thus constitute structurally (and also likely functionally) different species on the trypomastigote coat.

### Over-expression of TSSA does not affect epimastigote growth and differentiation

**Fig 2:**
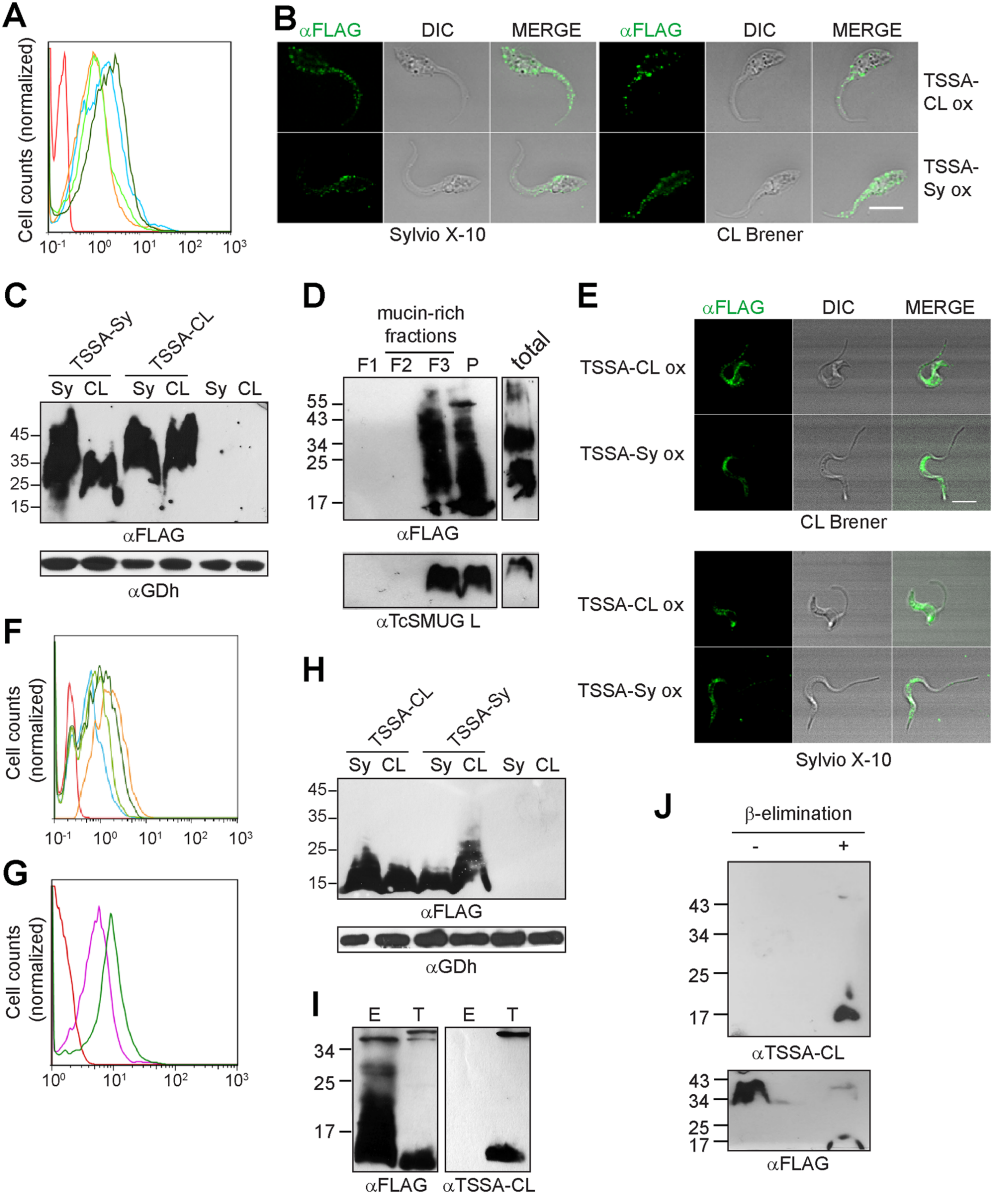
Over-expression of TSSA variants along the *T*. *cruzi* life cycle. **A, F** and **G)** Transgenic, non-permeabilized epimastigotes (panel A) or trypomastigotes (panels F and G) were labeled with polyclonal anti-FLAG antibodies (panels A and F) or mouse TSSA-CL antiserum (panel G) and evaluated by flow cytometry. CL Brener parasites transfected with TSSA-Sy or TSSA-CL are shown in light blue and dark green, respectively, whereas Sylvio X-10 parasites transfected with TSSA-Sy or TSSA-CL are shown in orange and light green, respectively. Isotype labeling control is depicted in red. In panel G, wild type CL Brener trypomastigotes are shown in orange. In each case, representative data of at least 3 experiments with similar results are shown. **B** and **E)** Immunofluorescence assays of permeabilized CL Brener or Sylvio X-10 epimastigotes (panel B) or trypomastigotes (panel E) over-expressing TSSA-CL or TSSA-Sy revealed with anti-FLAG mAb. Scale bars = 10 μm. **C** and **H)** Total extracts of epimastigotes (panel C) or trypomastigotes (panel H) from Sylvio X-10 (Sy) or CL Brener (CL) stocks either wild type or over-expressing the indicated molecule were probed with anti-FLAG mAb or *T*. *cruzi* glutamate dehydrogenase (GDh) antiserum. **D)** Butan-1-ol extraction analysis of CL Brener TSSA-CL ox epimastigotes. Fractions were obtained and named according to Materials and Methods, fractionated in SDS-PAGE and probed with anti-FLAG mAb or TcSMUG L antiserum. **I)** Total parasite extracts of CL Brener TSSA-CL ox epimastigotes (E) or trypomastigotes (T) were fractionated on to anti-FLAG–Sepharose and the retained fraction was probed by Western blot. **J)** Extracts of CL Brener TSSA-CL ox epimastigotes were subjected (+) or not (-) to ß-elimination and probed by Western blot with TSSA-CL antiserum or anti-FLAG mAb. Relative molecular mass markers (in kDa) are indicated.

In a previous work, we had already developed transgenic epimastigote lines over-expressing different TSSA variants with apparently contrasting features, termed TSSA-CL and TSSA-Sy [31]. Unfortunately, these transgenic lines were generated on the Adriana strain background, which did not undergo significant metacyclogenesis *in vitro* and hence precluded us to assess the impact of TSSA over-expression on trypomastigotes. To circumvent this limitation we attempted to develop TSSA over-expressing (TSSA ox) parasite lines on additional parental backgrounds. Constructs bearing FLAG-tagged TSSA variants were therefore sub-cloned into the *pTEX omni* vector and independently transfected into CL Brener (TcVI) epimastigotes. To expand our analysis, we also transfected these constructs into the Sylvio X-10 clone, comprised within the TcI *T*. *cruzi* phylogenetic group. As previously observed in the Adriana strain [31], TSSA ox epimastigotes showed no significant morphological or growth differences in comparison with parental, wild type parasites (S2 Fig). Expression level, surface localization and distribution of TSSA molecules was assessed by flow cytometry (Fig 2A) and confocal microscopy-based assays (Fig 2B).

As observed in trypomastigotes (see Fig 1A), TSSA ox epimastigotes from both genetic backgrounds bore a punctate pattern over their entire periphery, including the cell body and the flagellum (Fig 2B). Western blot analysis revealed a FLAG-reactive smear (~25–45 kDa) for either TSSA variant in transgenic epimastigotes (Fig 2C). This pattern of migration on SDS-PAGE clearly differed to that displayed by trypomastigote TSSA (see Fig 1B–1D), suggesting differences in the post-translational processing of this molecule along the parasite life cycle. Indeed, and at variance with trypomastigote-expressed TSSA (see Fig 1D), butan-1-ol extraction experiments supported extensive glycosylation of epimastigote-expressed TSSAs (Fig 2D). Part of FLAG-reactive species could be detected in the mucin-rich fraction F3; thus very similar to the extraction pattern obtained for the epimastigote-restricted TcSMUG L mucin-type products assayed in parallel (Fig 2D) [43].

*In vitro* assays revealed that TSSA ox epimastigotes display quite similar metacyclogenesis rates as compared to the corresponding parental lines (S3 Fig). Epimastigote cultures enriched in metacyclic trypomastigotes forms from every transgenic line were therefore used to infect Vero cell monolayers *in vitro* and, after several rounds of infection, mammal-dwelling forms of the parasite were obtained from culture medium. TSSA ox trypomastigotes were sorted by standard procedures and subjected to different biochemical and phenotypic analyses. Microscopy-based and flow-cytometry assays indicated that FLAG-tagged TSSAs accumulated at roughly similar levels on the surface of trypomastigotes from both genetic backgrounds (Fig 2E and 2F). As shown for native molecules (Fig 1A), FLAG-tagged TSSAs were not evenly spread along the trypomastigote membrane but following a rather patchy distribution (Fig 2E). To estimate the extent of TSSA over-expression in transgenic trypomastigotes, comparative flow cytometry experiments were carried out on CL Brener wild type and TSSA-CL-ox lines. In this case, parasites were labeled with a TSSA-CL antiserum, revealing that the TSSA-CL-ox line expressed ~30% more TSSA-CL than the parental line (Fig 2G). This experiment could not be performed in Sylvio X-10 parasites due to lack of an appropriate TSSA-Sy antiserum. Interestingly, Western blot experiments revealed that FLAG-tagged TSSAs were expressed as a less diffused band of ~15–20 kDa band on TSSA ox trypomastigotes of both genetic backgrounds (Fig 2H), hence quite similar to the native molecules (Fig 1B–1D) and distinct of the TSSAs of over-expressing epimastigotes (Fig 2C). The limited number of trypomastigotes yielded by TSSA ox lines (see below) precluded us to carry out detailed biochemical characterizations of FLAG-tagged TSSAs. However, anti-FLAG immunoprecipitation assays followed by Western blot using the TSSA-CL antiserum revealed a contrasting recognition profile for epimastigote- vs trypomastigote-expressed products (Fig 2I), further supporting a differential processing for TSSA on distinct developmental forms of the parasite. Moreover, release of *O*-glycans by ß-elimination led to a shift in the migration pattern of the TSSA-CL expressed by epimastigotes (from a ~34–45 kDa smear to a ~17 kDa species), and to its recognition by the TSSA-CL antiserum (Fig 2J).

### TSSA over-expression promotes trypomastigote-to-amastigote transformation

**Fig 3:**
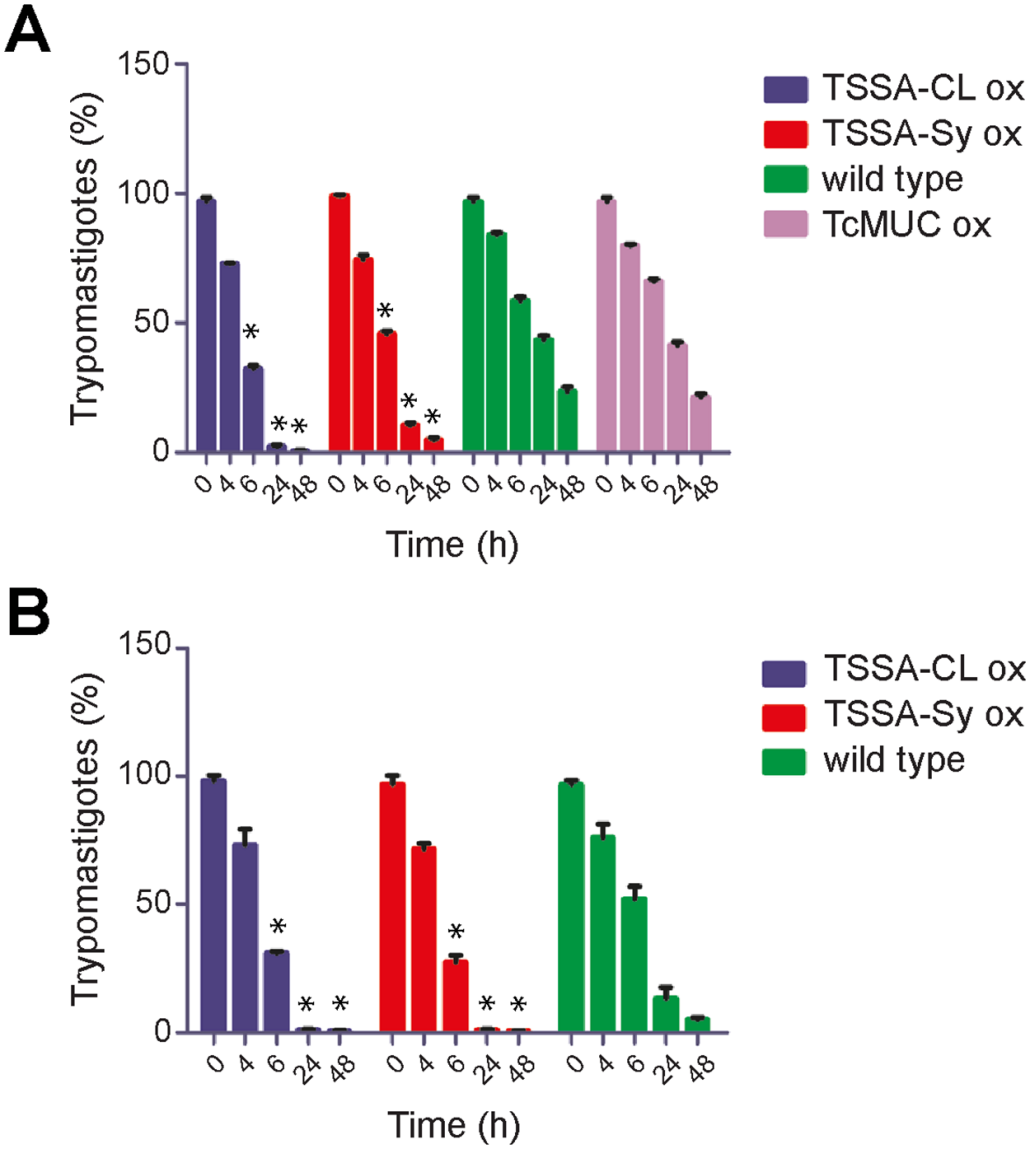
Over-expression of TSSA variants improves trypomastigote-to-amastigote transformation kinetics in *T*. *cruzi*. Purified, CL Brener (panel A) or Sylvio X-10 (panel B) trypomastigotes (5 x 10^6^) of the indicated line were incubated in MEM at pH7, without serum. Samples were taken at different time-points, fixed, and total number of trypomastigotes and amastigotes were counted directly under the light microscope. For each sample, at least 300 parasites were counted and trypomastigotes were expressed as % of total parasites. The results are the average of 3 independent experiments. Asterisks denote significant differences (*P* < 0.05) to wild type parasites using *t*-Student test.

A much higher ratio of extracellular amastigotes to trypomastigotes was consistently observed in the supernatant of cells infected with TSSA ox parasites as compared with parental, wild type lines. This bias suggested an imbalance in parasite infectivity, intracellular growth and/or trypomastigote-to-amastigote differentiation. To evaluate the latter issue, we carried out extracellular amastigogenesis assays on CL Brener lines. As shown in Fig 3A, the transformation process was completed after 24 h of incubation at pH7 for both TSSA-CL and TSSA-Sy ox trypomastigotes whereas the parental line took > 48 h to assess.

To rule out a possible non-specific effect of transfection and/or protein over-expression on this phenotype, we evaluated in parallel the kinetics of transformation of CL Brener TcMUC ox trypomastigotes. These parasites were transfected with a *pTEX omni* vector bearing a FLAG-tagged, canonical TcMUC product [41], selected and differentiated as TSSA ox lines. In contrast to TSSA ox lines, however, TcMUC ox trypomastigotes displayed similar kinetics of transformation than the parental, wild type line (Fig 3A). Similar results were obtained for TSSA ox trypomastigotes in the Sylvio X-10 background (Fig 3B), or when trypomastigote-to-amastigote transformation was evaluated at pH5 morphologically or by means of an amastigote-specific antibody (S4 Fig). No significant differences in the viability of distinct parasite lines along the amastigogenesis experiments were observed (S4 Fig). Together, these findings suggest that over-expression of TSSA leads to an exacerbated amastigogenesis.

### TSSA-CL but not TSSA-Sy is involved in trypomastigote recognition of mammalian cells

**Fig 4:**
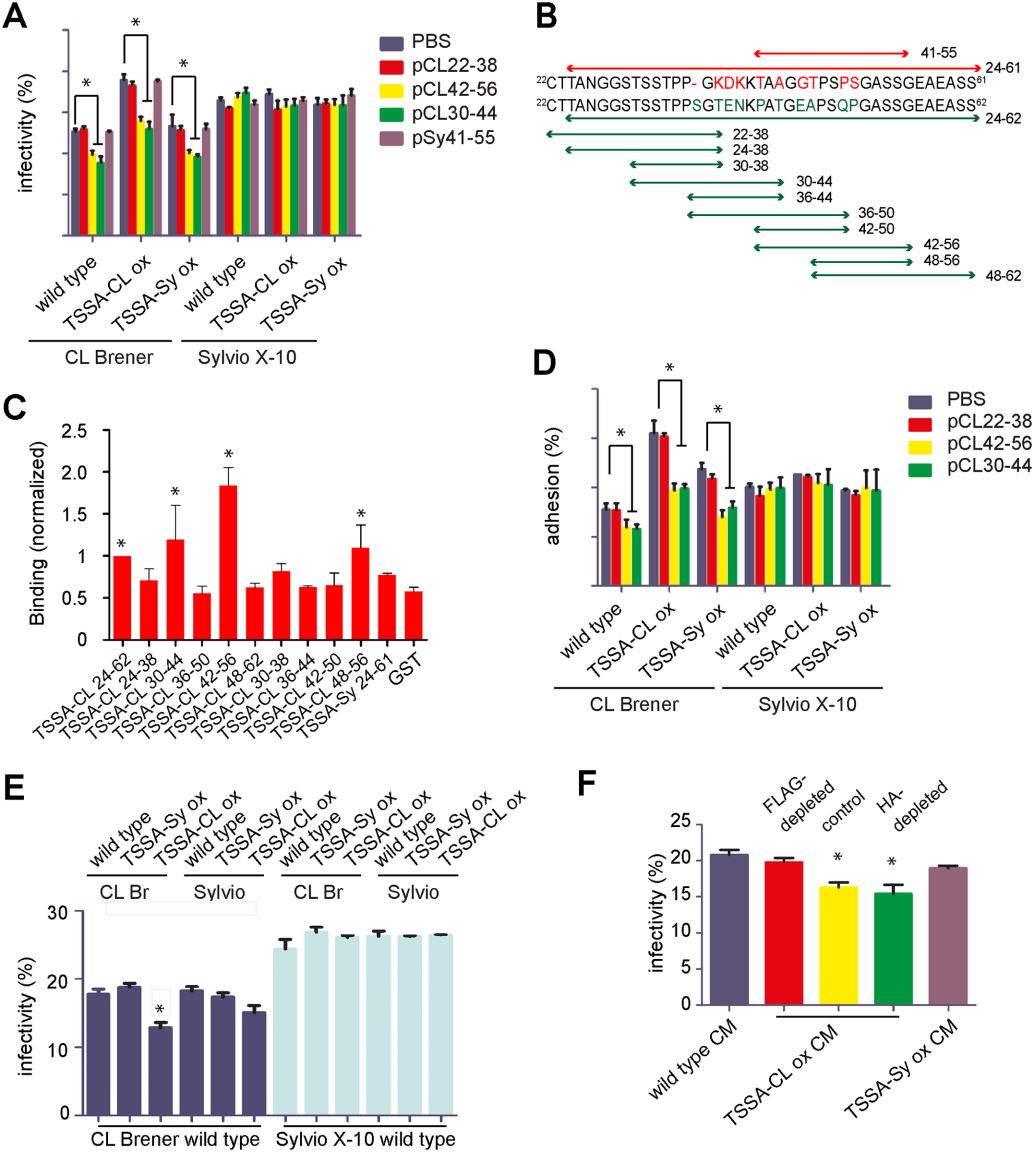
TSSA-CL, but not TSSA-Sy, is involved in trypomastigote internalization. **A** and **D)** Infection (panel A) or adhesion (panel D) rates of transgenic or wild type trypomastigote lines towards Vero cell monolayers were measured in presence of 100 μg/mL of the indicated peptide or PBS as control. Vero cells grown on 24-well culture plates were added with 1 or 2 x 10^5^ Sylvio X-10 or CL Brener trypomastigote forms (with up to 5% of contaminant amastigote forms), respectively. After 3 h of incubation at 4°C (panel D) or at 37°C (panel A), cells were washed with PBS to remove non-attached parasites, fixed with PBS-PFA immediately (panel D) or after additional 36 h incubation at 37°C (panel A) and processed for Indirect Immunofluorescence assay. In all experiments, the number of infected cells was determined in a total of at least 1,000 DAPI-stained cells. Data are expressed as mean values ± SD of 3 independent experiments, each one performed in duplicate. B) Schematic representation of TSSA-Sy (above) and TSSA-CL (below) sequences expressed as GST-fusion molecules or synthetic peptides, and the residues spanned by each construct (numbers indicate amino acid positions in each sequence relative to the initial Met). Variations between TSSAs and sequences derived thereof are indicated in red (for TSSA-Sy) and green (for TSSA-CL). **C)** GST-fusion proteins spanning TSSA-CL deletion variants were added to HeLa cells and binding was assessed by means of a monoclonal anti-GST antibody followed by a colorimetric method. In every assay, recombinant GST and GST-TSSA-Sy^24-61^ proteins were used as negative control whereas GST-TSSA-CL^24-62^ was used as positive control. Reactivity for each protein was normalized to GST-TSSA-CL^24-62^ and mean ± SD was calculated from 3 independent assays. Significant differences between the indicated population and GST means (*P* < 0.05 ANOVA followed by Dunnet’s correction) were denoted with an asterisk. **E)** Infection rates of wild type trypomastigote lines (indicated below) towards Vero cell monolayers were measured in presence of different transgenic or wild type trypomastigote lines in the upper chamber of the trans-well (indicated above). **F)** Infection rates of wild type CL Brener trypomastigotes in the presence of conditioned medium (CM) of the indicated trypomastigote line were measured as described above. When indicated, CM was FLAG- or Hemmaglutinin-depleted by affinity chromatography before the flow-through fraction being incubated with wild type CL Brener trypomastigotes. In panels A, C, D and E asterisks denote significant differences (*P* < 0.05) to the corresponding control values using *t*-Student test.

We next performed *in vitro* infection experiments with transgenic or parental trypomastigotes. The infection rate was determined 36 h afterwards by direct counting of infected and non-infected cells. In the CL Brener background, TSSA-CL ox parasites displayed an enhanced infectivity (~17%) as compared to those over-expressing TSSA-Sy or parental controls (Fig 4A). No significant differences between transgenic and parental lines were however observed when the number of parasites/infected cell was evaluated (S5 Fig), suggesting that over-expression of TSSA does not affect parasite cell cycle and/or intracellular growth.

To further address the specificity of these results, we aimed at carrying out similar infection assays in the presence of exogenously added TSSA-derived peptides (indicated in Fig 4B). To identify the host cell binding sequence(s) in TSSA-CL we followed a deletion mapping analysis using a panel of GST-fusion molecules. Host cell binding of each purified recombinant protein was assessed as described [31]. As shown in Fig 4C, most of TSSA-CL-derived sequences had negligible binding activity, similar to that of GST or a GST-fusion spanning residues 24 to 61 of TSSA-Sy (GST-TSSA-Sy^24-61^) used as negative controls [31]. Conversely, GST-TSSA-CL^42-56^ and to a lower extent its included sequence GST-TSSA-CL^48-56^ and GST-TSSA-CL^30-44^ showed significant binding to cultured cells (Fig 4B and 4C), similar to what has been originally described for GST-TSSA-CL^24-62^ [31]. Moreover, GST-TSSA-CL^42-56^ presented improved binding than GST-TSSA-CL^24-62^ (Fig 4C), suggesting structural constraints imposed by other residues in the latter protein that partially impair its interaction with cell surface ligand(s).

Importantly, the improved infectivity of TSSA-CL ox trypomastigotes could be specifically counteracted by preincubation with peptides pCL30-44 or pCL42-56 (Fig 4A). Peptide pCL22-38, which did not show host cell binding properties (see GST-TSSA-CL^24-38^ in Fig 4C), or peptide pSy41-55, which encompasses the corresponding sequence of pCL42-56 in the non-adhesive TSSA-Sy protein (Fig 4B) did not affect infectivity of TSSA-CL ox parasites when assayed at the same concentration (Fig 4A). Neither peptide affected parasite viability under the assayed conditions, as revealed by propidium iodide uptake (S5 Fig).

Similar experiments were carried out in order to determine parasite adhesion rates. In line with above results, TSSA-CL ox parasites showed increased adhesion to cultured cells as compared to wild type or TSSA-Sy ox ones, and this effect could also be specifically counteracted by peptides derived from the adhesive motifs of TSSA-CL but not with control peptides or PBS (Fig 4D). Overall, our data show a strict correlation between peptide ability to bind to host cells and to inhibit TSSA-driven adhesion (and therefore infectivity) of CL Brener trypomastigotes. These findings strongly suggest that recombinant and native TSSA-CL display highly similar specificities. In the Sylvio X-10 background, and in sharp contrast to above results, over-expression of neither TSSA variant had a significant impact on trypomastigote infectivity and/or adhesion (Fig 4A and 4D).

Considering that TSSA-CL is actively secreted to the medium (Fig 1E), we next evaluated whether it may also modulate infectivity of trypomastigotes *in trans*. To that end, the infectivity of trypomastigotes was assessed as before, though in the presence of additional parasites placed on the upper chamber of trans-well microplates. As shown in Fig 4E, a significant decrease in the infection rate was observed when wild type CL Brener trypomastigotes were exposed to the CM of CL Brener TSSA-CL ox parasites as compared to those exposed to the CM of wild type or TSSA-Sy ox counterparts. A similar trend was observed when CL Brener trypomastigotes were exposed to the CM of different Sylvio X-10 parasite lines. In this case, however the decrease in the infection rate of wild type CL Brener trypomastigotes exposed to the CM of Silvio X-10 TSSA-CL ox parasites was not of statistical significance (Fig 4E). The infectivity rate of Sylvio X-10 trypomastigotes, on the other hand, was not affected by the CM of every tested trypomastigote line (Fig 4E). A complete profile of the CM from Sylvio X-10 and CL Brener trypomastigotes, as well as from different transgenic lines is shown in S6 Fig.

We next assessed the infectivity of wild type CL Brener trypomastigotes directly added with purified CM of different CL Brener lines. As shown in Fig 4F, the CM from TSSA-CL ox trypomastigotes had a significant inhibitory effect as compared to the CM of TSSA-Sy ox or wild type counterparts. The effect exerted by the CM of TSSA-CL ox parasites could be depleted by FLAG-affinity chromatography (Fig 4F), strongly suggesting that FLAG-tagged TSSA-CL molecules, likely associated to actively secreted MVs (see Fig 1F), constitute the ‘soluble factor’ underpinning this inhibition. Altogether, these results strongly support a direct involvement of TSSA-CL, via adhesive motifs of peptidic nature in the interaction between CL Brener trypomastigotes with the target cell prior to parasite internalization.

## Discussion

Antigenicity mapping experiments [33,47] and biochemical data [31] converged in suggesting the exposition of peptidic moieties on surface-associated TSSA molecules, which was at odds with *TSSA* being part of the *TcMUC* gene family. In the first part of this work, we undertook a series of biochemical approaches to directly address this issue. Microscopy-based experiments showed that TSSA-CL, as previously reported for other trypomastigote surface markers [19], does not co-localize with sialylated tGPI-mucins. The punctate pattern observed for TSSA-CL builds upon our hypothesis of the trypomastigote membrane as a highly organized structure made up of multiple and discrete nanoscale domains bearing different protein composition [48]. Further affinity fractionation experiments and whole-parasite butan-1-ol partition assays definitely established that TSSA and tGPI-mucins are completely different species. At variance with tGPI-mucins, which undergo extensive *O*-glycosylation and terminal sialylation to fulfill their main protective and immunomodulatory roles [11], TSSA seems to be displayed as a hypo-glycosylated (or non-glycosylated at all) molecule on the trypomastigote surface. Although this proposal is based on experimental data and seems to have a functional correlate (see below), a detailed structural analysis of TSSA would be required to definitely address this issue.

Host cell binding assays and the use of trypomastigote transgenic lines support that exposition of TSSA ‘naked’ peptide sequences is critical for its engagement of yet unidentified host cell surface receptor(s) prior to trypomastigote effective internalization. As shown, at least 2 TSSA-CL-derived synthetic peptides (from residue 42 to 56 and from residue 30 to 44) displaying adhesion properties to cultured cells *in vitro*, were able to interfere with or partially block CL Brener trypomastigote-host cell interactions, indicating that these peptidic motifs are involved in the receptor pairing of the native, surface-associated TSSA-CL molecule *in vivo*. Sequence polymorphisms between TSSA-CL and TSSA-Sy variants were shown to be focused on the central region of this molecule (from residue 36 to 51), and likely explain the significant differences in their host cell binding properties [34,36,38]. Taking into account that TSSA-CL likely contributes to trypomastigote-host cell binding, it may be also speculated that its aggregation in discrete domains along the surface coat increases the avidity of its mediated interactions, hence providing the trypomastigote with a solid grasp to the target cell. Interestingly, anti-TSSA-CL antibodies elicited by Chagasic patients are preferentially directed towards linear epitopes contained within the herein identified adhesive motifs of TSSA-CL and are thus expected to have a direct detrimental effect on parasite infectivity [33,47].

In the second part of this work, we generated transgenic epimastigote lines over-expressing TSSA variants to get further insights into its *in vivo* functional role(s). Independently of the recipient strain and the transfected construct, TSSA over-expression led to the surface accumulation and ‘patchy’ distribution of a FLAG-tagged product, which does not affect epimastigote growth and/or differentiation into metacyclic forms. Curiously, epimastigote-expressed TSSAs undergo mucin-type glycosylation. This kind of ‘aberrant’ processing has been previously observed in TSSA ox epimastigotes from the Adriana strain [31], suggesting it is a common feature of this parasite stage, likely attributed to its particular profiling of glycosyl transferases [18]. Upon transformation into trypomastigotes, and in strict accordance to previous recombinant protein binding data [31], CL Brener trypomastigote forms over-expressing TSSA-CL, but not TSSA-Sy, show significantly improved adhesion and infectivity towards non-macrophagic cells. Even though we cannot formally rule out the possible contribution of additional surface adhesin(s) whose expression/processing may become deregulated in transgenic parasites, the counteracting effect of peptides spanning TSSA-CL adhesion motifs allowed us to nail down this phenotype to TSSA-CL.

Over-expression of TSSA-CL does not have a significant impact on the infectivity and/or adhesion of Sylvio X-10 trypomastigotes, which is in principle difficult to reconcile with our main hypothesis. However, the fact that Sylvio X-10 parasites (not encoding for an adhesive TSSA variant) are nonetheless adhesive/infectious in our *in vitro* system indicates that target cell recognition/invasion capabilities of these parasites rely on a different subset of surface receptor(s). As mentioned, *T*. *cruzi* trypomastigotes bear a huge repertoire of apparently redundant adhesins [5–7]. As part of the compensatory mechanisms, we hypothesize that some variants of these adhesion molecules showing improved expression/function may have been selected for in Sylvio X-10 parasites. Indeed, several quantitative and/or qualitative differences of surface molecules between *T*. *cruzi* strains, some of which are likely associated to parasite phenotypic variations, have been described [49–52]. The differential ‘surface coat environment’ of Sylvio X-10 trypomastigotes may be responsible for buffering the otherwise net increase in trypomastigote infectiveness caused by over-expression of TSSA-CL. Alternatively, it may be speculated that TSSA-CL undergoes a particular processing in Sylvio X-10 trypomastigotes. However, the facts that this molecule is recognized by TSSA-CL antiserum (S7 Fig) and that it is able to partially block the infectivity of CL Brener parasites *in trans* (Fig 4E) argue against this possibility.

In addition to its role in trypomastigote-host cell interactions, we show that TSSA ox trypomastigotes independently of the variant and parasite genetic background, display exacerbated amastigogenesis. The use of control trypomastigotes over-expressing a distinct GPI-anchored molecule undergoing similar intracellular processing and trafficking pathways such as TcMUC ox [41] indicate that this phenotype cannot be related to a global secretory system depression and/or saturation of transport mechanisms. The molecular and cellular basis underlying the putative link between TSSA and amastigogenesis remain to be addressed.

In summary, we have shown that in spite of being encoded by a *TcMUC*-like gene and being co-expressed on the trypomastigote coat, TSSA is neither structurally not functionally related to tGPI-mucins. Our data indicates that this molecule plays an important role not only on the infectivity of trypomastigotes but also on the phenotypic variability displayed by *T*. *cruzi* strains [37], and strongly support this molecule as an excellent candidate for molecular intervention and/or vaccine development in Chagas disease.

## Supporting information

S1 Fig Profile of recognition of TSSA-CL and TcMUC antisera. Western blot of total lysates from *T*. *cruzi* (CL Brener clone) epimastigotes (E), cell-derived trypomastigotes (T), and amastigotes (A). Approximately 2 x 10^7^ parasites were loaded in each lane of a SDS-PAGE gel and assayed by Western blotting with the indicated antiserum. A faint ~40 kDa band of unknown identity and not consistently observed in every mouse TSSA-CL antiserum-developed Western blot is denoted with an asterisk. Relative molecular mass markers (in kDa) are indicated.
(TIF)

S2 Fig Growth curves of TSSA ox parasites. Wild type (black), TSSA-CL ox (green) or TSSA-Sy ox (orange) epimastigotes of the CL Brener (solid lines) or Sylvio X-10 (dotted lines) clones were seeded at a density of 1 x 10^6^ parasites per mL in BHT 10% FCS without G418 and counted at the indicated time-points in a Neubauer chamber.
(TIF)

S3 Fig Metacyclogenesis curves of TSSA ox parasites. Exponentially growing epimastigotes (2 x 10^6^ per mL) indicated as in legend to S2 Fig were diluted in BHT 10% and maintained at 28°C without agitation. Samples were taken and processed as above at the indicated time-points. For each sample, at least 150 parasites were counted and discriminated by morphology under light microscope, and metacyclic forms were expressed as % of total parasites.
(TIF)

S4 Fig Over-expression of TSSA variants improves trypomastigote-to-amastigote transformation kinetics in *T*. *cruzi*. **A-D)** Purified, CL Brener (panels A and C) or Sylvio X-10 (panels B and D) trypomastigotes (5 x 10^6^) of the indicated line were incubated in MEM at pH5, without serum. Samples were taken at different time-points, fixed, and total number of trypomastigotes and amastigotes were counted directly under the light microscope (panels A and B) or upon indirect immunofluorescence assays revealed with an amastin antiserum (1:500 dilution, panels C and D). For each sample, at least 300 parasites were counted and trypomastigotes were expressed as % of total parasites. The results are the average of 3 independent experiments. Asterisks denote significant differences (*P* < 0.05) to wild type parasites using *t*-Student test. **E)** Representative image of CL Brener parasites processed for immunofluorescence using mouse amastin antiserum. White arrowheads point to strongly labeled amastigote forms whereas cyan arrowheads indicate cell-derived trypomastigotes. Reactivity of the latter forms range from negative to weak. **F)** Viability of parasites from the indicated line along the amastigogenesis assays was assessed by propidium iodide uptake.
(TIF)

S5 Fig Over-expression of TSSA variants does not affect intracellular growth of *T*. *cruzi*. **A)** Vero cell monolayers were infected with different transgenic or wild type trypomastigote lines as indicated in legend to Fig 4 and the number of parasites per infected cell was determined in a total of at least 1,000 DAPI-stained cells. Data are expressed as mean values ± SD of 3 independent experiments performed in duplicate. **B)** Viability of CL Brener cell-derived trypomastigote forms incubated with the indicated peptide was assessed by propidium iodide uptake.
(TIF)

S6 Fig Over-expression of TSSA variants does not affect the secretion profile of cell-derived trypomastigotes. Conditioned medium from cell-derived trypomastigote forms (~1 x 10^8^) of the indicated parasite lines were fractionated onto SDS-PAGE followed by silver staining. Molecular mass markers (in kDa) are indicated.
(TIF)

S7 Fig TSSA-CL is not expressed as a mucin-type molecule in Sylvio X-10 trypomastigotes. Total parasite extracts of Silvio X-10 TSSA-CL ox epimastigotes (E) or trypomastigotes (T) were probed by Western blot using the mouse TSSA-CL antiserum. Molecular mass markers (in kDa) are indicated.
(TIF)
