# Supplementary figures and images for: The Trypomastigote Small Surface Antigen (TSSA) regulates Trypanosoma cruzi infectivity and differentiation
Source: PLoS Negl Trop Dis. 2017 Aug 11;11(8):e0005856. doi: 10.1371/journal.pntd.0005856 (PMC5568413; doi:10.1371/journal.pntd.0005856)

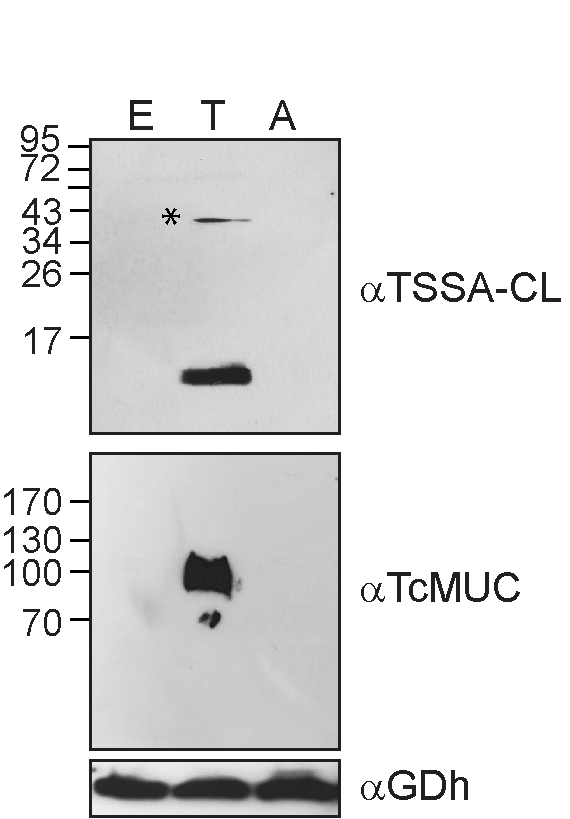

Supplement: S1 Fig — Western blot of total lysates from T. cruzi (CL Brener clone) epimastigotes (E), cell-derived trypomastigotes (T), and amastigotes (A). Approximately 2 x 10^7^ parasites were loaded in each lane of a SDS-PAGE gel and assayed by Western blotting with the indicated antiserum. A faint ~40 kDa band of unknown identity and not consistently observed in every mouse TSSA-CL antiserum-developed Western blot is denoted with an asterisk. Relative molecular mass markers (in kDa) are indicated. (TIF) [file pntd.0005856.s001.tif]

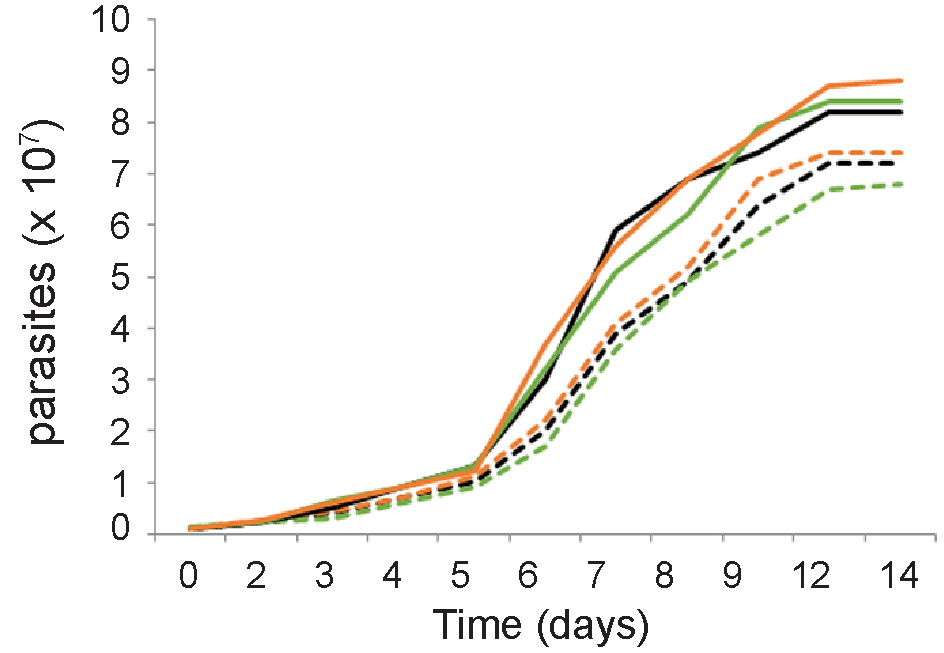

Supplement: S2 Fig — Wild type (black), TSSA-CL ox (green) or TSSA-Sy ox (orange) epimastigotes of the CL Brener (solid lines) or Sylvio X-10 (dotted lines) clones were seeded at a density of 1 x 10^6^ parasites per mL in BHT 10% FCS without G418 and counted at the indicated time-points in a Neubauer chamber. (TIF) [file pntd.0005856.s002.tif]

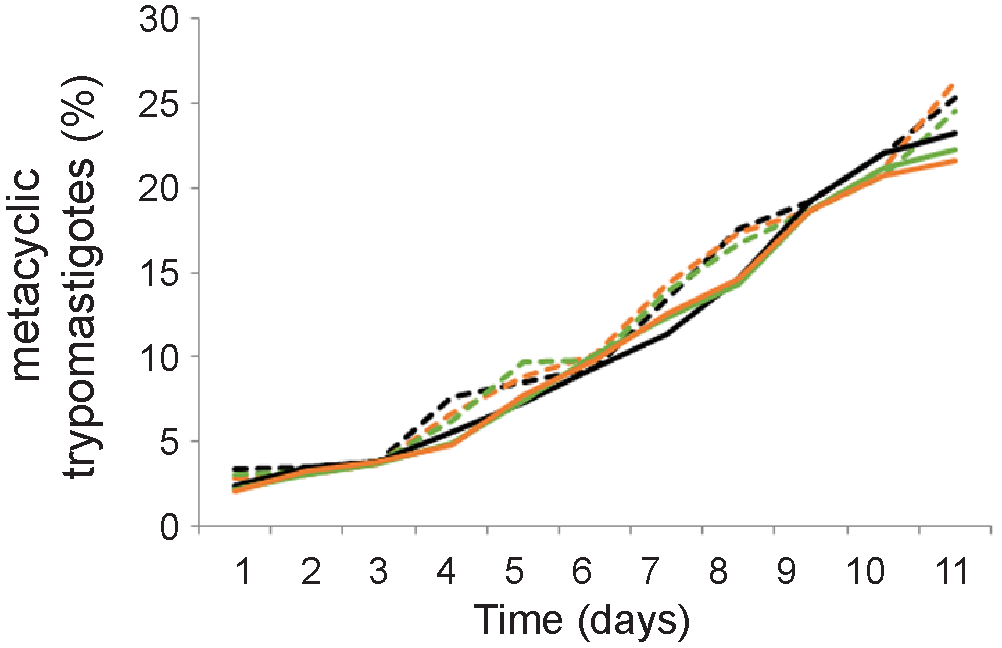

Supplement: S3 Fig — Exponentially growing epimastigotes (2 x 10^6^ per mL) indicated as in legend to S2 Fig were diluted in BHT 10% and maintained at 28°C without agitation. Samples were taken and processed as above at the indicated time-points. For each sample, at least 150 parasites were counted and discriminated by morphology under light microscope, and metacyclic forms were expressed as % of total parasites. (TIF) [file pntd.0005856.s003.tif]

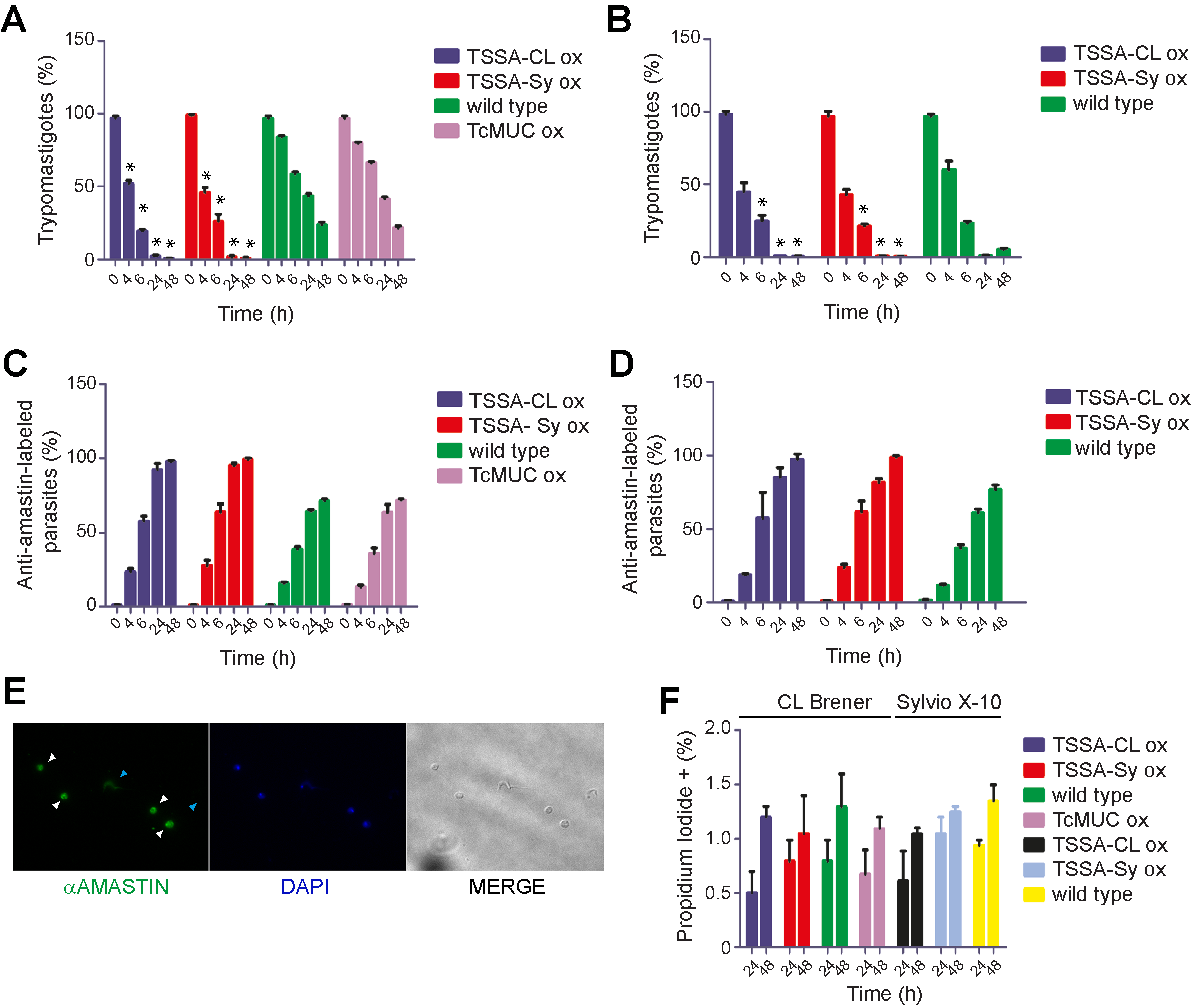

Supplement: S4 Fig — A-D) Purified, CL Brener (panels A and C) or Sylvio X-10 (panels B and D) trypomastigotes (5 x 10^6^) of the indicated line were incubated in MEM at pH5, without serum. Samples were taken at different time-points, fixed, and total number of trypomastigotes and amastigotes were counted directly under the light microscope (panels A and B) or upon indirect immunofluorescence assays revealed with an amastin antiserum (1:500 dilution, panels C and D). For each sample, at least 300 parasites were counted and trypomastigotes were expressed as % of total parasites. The results are the average of 3 independent experiments. Asterisks denote significant differences (P < 0.05) to wild type parasites using t-Student test. E) Representative image of CL Brener parasites processed for immunofluorescence using mouse amastin antiserum. White arrowheads point to strongly labeled amastigote forms whereas cyan arrowheads indicate cell-derived trypomastigotes. Reactivity of the latter forms range from negative to weak. F) Viability of parasites from the indicated line along the amastigogenesis assays was assessed by propidium iodide uptake. (TIF) [file pntd.0005856.s004.tif]

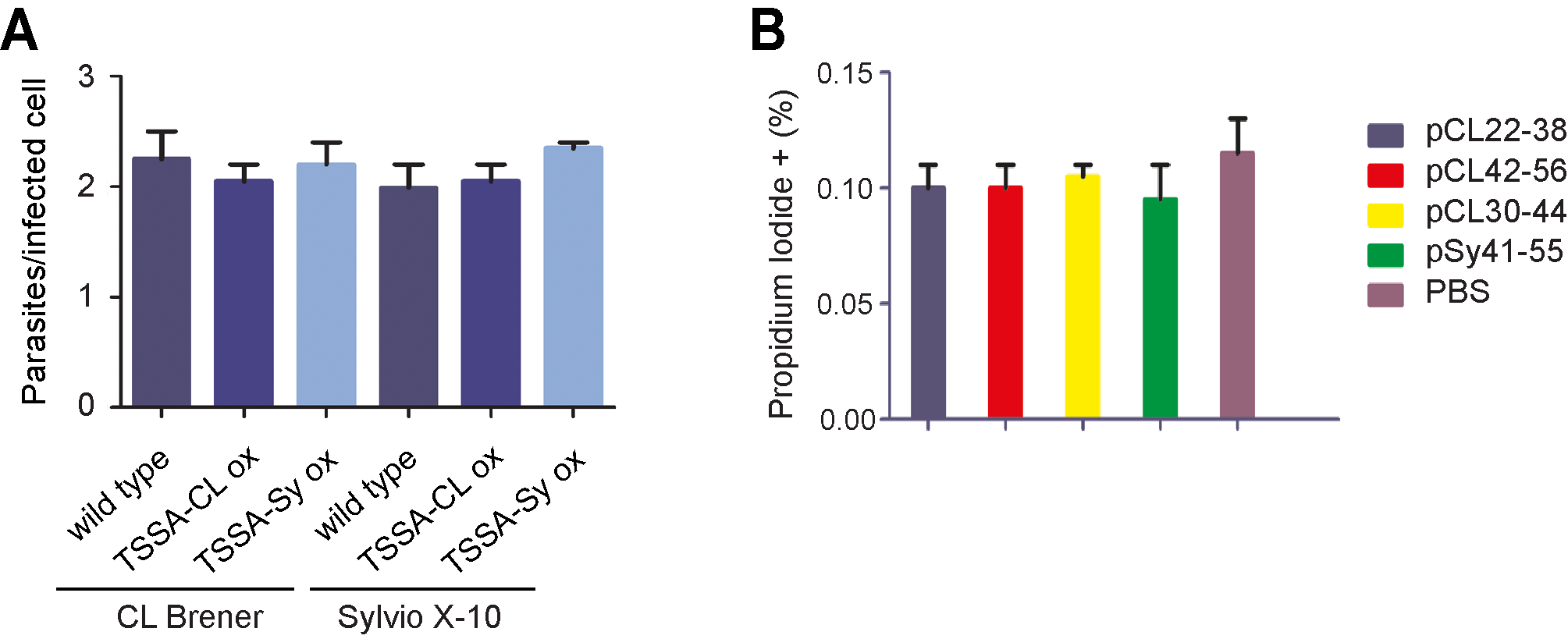

Supplement: S5 Fig — A) Vero cell monolayers were infected with different transgenic or wild type trypomastigote lines as indicated in legend to Fig 4 and the number of parasites per infected cell was determined in a total of at least 1,000 DAPI-stained cells. Data are expressed as mean values ± SD of 3 independent experiments performed in duplicate. B) Viability of CL Brener cell-derived trypomastigote forms incubated with the indicated peptide was assessed by propidium iodide uptake. (TIF) [file pntd.0005856.s005.tif]

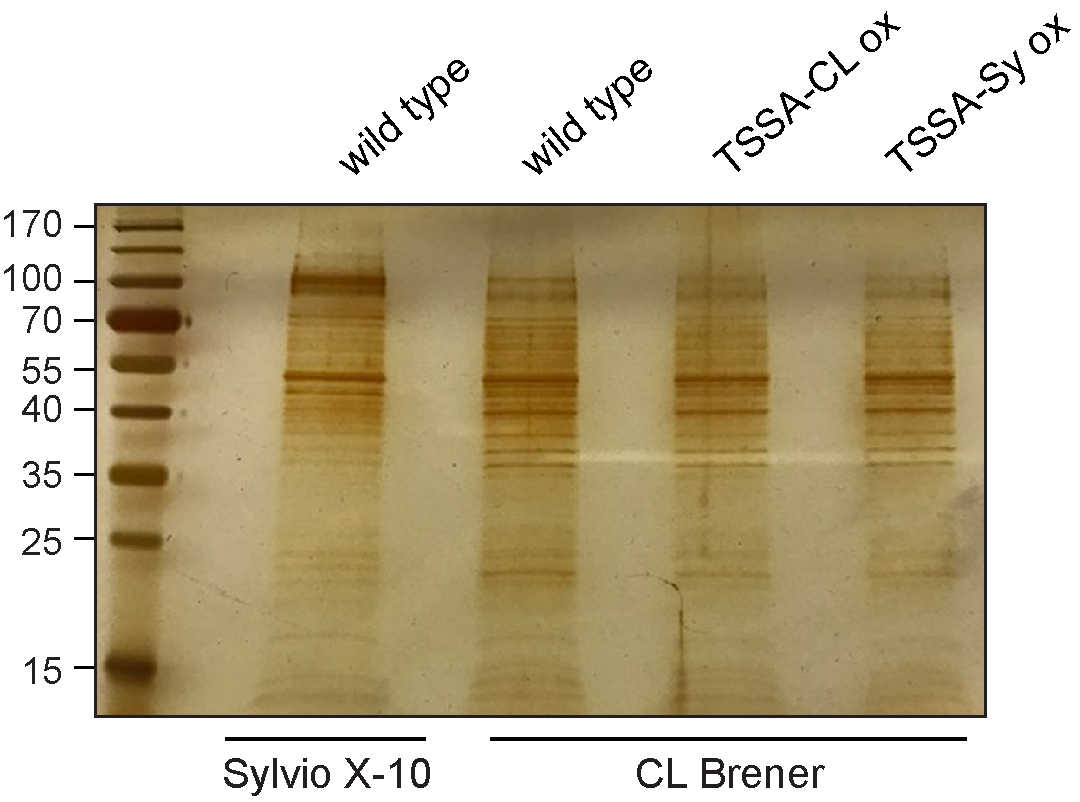

Supplement: S6 Fig — Conditioned medium from cell-derived trypomastigote forms (~1 x 10^8^) of the indicated parasite lines were fractionated onto SDS-PAGE followed by silver staining. Molecular mass markers (in kDa) are indicated. (TIF) [file pntd.0005856.s006.tif]

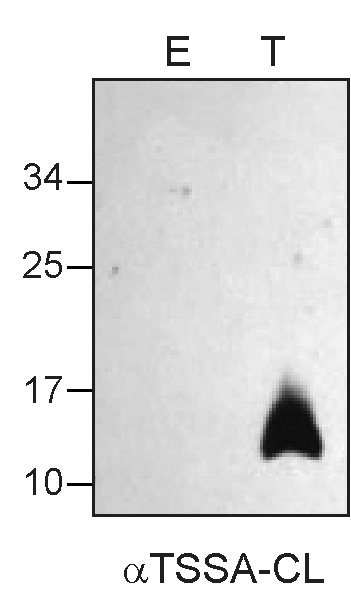

Supplement: S7 Fig — Total parasite extracts of Silvio X-10 TSSA-CL ox epimastigotes (E) or trypomastigotes (T) were probed by Western blot using the mouse TSSA-CL antiserum. Molecular mass markers (in kDa) are indicated. (TIF) [file pntd.0005856.s007.tif]
